# Supplementary material for: Responding to harvest failure: Understanding farmers coping strategies in the semi-arid Northern Ghana
Source: PLoS One. 2023 Apr 14;18(4):e0284328. doi: 10.1371/journal.pone.0284328 (PMC10104303; doi:10.1371/journal.pone.0284328)
Supplement: S1 Appendix — (DOCX) [file pone.0284328.s001.docx]

Table A1. Number of households selected per community and district

| No. | Community  Surveyed | Kassena-NE District | Kassena-NW District | Bolgatanga Municipal | Talensi District | Nabdam District | Total |
| --- | --- | --- | --- | --- | --- | --- | --- |
| 1 | Doba Gayingo | 10 |  |  |  |  | 10 |
| 2 | Kologu Awuyabisi | 10 |  |  |  |  | 10 |
| 3 | Gia-Nangawao | 15 |  |  |  |  | 15 |
| 4 | Manyoro-Wura | 10 |  |  |  |  | 10 |
| 5 | Korania-Shrine | 10 |  |  |  |  | 10 |
| 6 | Punyoro | 15 |  |  |  |  | 15 |
| 7 | Sirigu-Basengo |  | 14 |  |  |  | 14 |
| 8 | Mirigu-Gonum |  | 15 |  |  |  | 15 |
| 9 | Kandiga-Kurugu |  | 15 |  |  |  | 15 |
| 10 | Katiu Asasong |  | 15 |  |  |  | 15 |
| 11 | Zaare Avombisi |  |  | 22 |  |  | 22 |
| 12 | Sherigu-Kumblingu |  |  | 24 |  |  | 24 |
| 13 | Gambibgo-Azoabisi |  |  | 22 |  |  | 22 |
| 14 | Zuarungu Dachio |  |  | 22 |  |  | 22 |
| 15 | Baare Talaha |  |  |  | 10 |  | 10 |
| 16 | Baare Tengre |  |  |  | 10 |  | 10 |
| 17 | Koriga Zandoy Station |  |  |  | 10 |  | 10 |
| 18 | Datuku Zanwore |  |  |  | 10 |  | 10 |
| 19 | Nangodi Kalini |  |  |  |  | 10 | 10 |
| 20 | Nangodi Nyoboka |  |  |  |  | 10 | 10 |
| 21 | Zanlerigu Gaah (Tindo) |  |  |  |  | 10 | 10 |
| 22 | Namolgo Tewoog |  |  |  |  | 10 | 10 |
|  | Total | 70 | 59 | 90 | 40 | 40 | 299 |

Source: Authors (based on household survey data)
