# Supplementary material for: Responding to harvest failure: Understanding farmers coping strategies in the semi-arid Northern Ghana
Source: PLoS One. 2023 Apr 14;18(4):e0284328. doi: 10.1371/journal.pone.0284328 (PMC10104303; doi:10.1371/journal.pone.0284328)
Supplement: S1 File — (DOCX) [file pone.0284328.s002.docx]

**Supporting information**

For the consumer/worker ratio in Table 1, the conversion factors for the consumer units are modified versions of the age range proposed by Runge-Metzger and Diehl (1993), while the conversion factors for the worker units are from Boansi *et al.,* (2017)

**References**

Runge-Metzger A, Diehl L. Farm household systems in Northern Ghana: A case study in farming systems-oriented research for development of improved crop production systems. Nyankpala Agricultural Research Station, Tamale, (Special Report No. 9). 1993

Boansi D, Tambo JA, Müller M. Analysis of farmers’ adaptation to weather extremes in West African Sudan Savanna. *Weather and Climate Extremes*. 2017; 16:1-13
