# Supplementary material for: Responding to harvest failure: Understanding farmers coping strategies in the semi-arid Northern Ghana
Source: PLoS One. 2023 Apr 14;18(4):e0284328. doi: 10.1371/journal.pone.0284328 (PMC10104303; doi:10.1371/journal.pone.0284328)
Supplement: Attachment — Submitted filename: Plosone_22.docx [file pone.0284328.s004.docx]

1. Authors trying to justify their study asserted that most studies concentrate on adaptation or adaptive strategies leaving out coping strategies. This might not be true as there a lot of studies that have looked at coping strategies in the area. Also, the variables measured by authors as coping strategies are the same measured in the studies, they are saying have concentrated on coping strategies. So it might be a matter of semantics. Therefore, authors need to revisit the problem statement and identify clearly what they are trying to do.

2. In the study area description, authors use statistics without making references to them (see line 326 and 327). These statistics are from other works and must be cited.

3. Methodology

The methodology has many flaws. First of all, in lines 273 and 274, another state that both qualitative and quantitative methods are used and yet, throughout the work, no qualitative method nor results presented. What qualitative data were collected? What qualitative method was used and how were the qualitative data analysed? The selection of the districts was skewed. All districts selected are in the central part of the region. With the exception of the Kasenas, the tribes occupying these districts virtually have the same cultural practices. However, the West (Bulsas) and the East (Kusasis and Mamprusis) are left out. Consideration of tribe/ethnicity is very important as culture affect the kind of adaptive strategies or coping strategies. Why did they concentrate on the middle? There should be a justification. Authors should note these East and West districts are the food basket of the region and any study of this kind should not leave them out. Also, the simple random and systematic selections are not described in detail. How was the simple random sampling done? How was the systematic Selection done? How many communities in each district was selected and why? Were these communities also selected by the simple random or systematic? How many farmers in each community were selected and why? All these questions need to be addressed in the methodology.

4. Results

#1. There are equations in the methods, but the results are not presented to reflect results from the equations. Most of the presentations can be done with cross-tabulation and that will show clear relationships than the results presented.

#2. Authors report that between 2005 and 2014, the year 2014 was the year with most crop failure. I turn to disagree with this because crop failure depends on many factors including climatic events such as floods and droughts. In 2007, the entire northern Ghana recorded the worst floods leading to the worse crop failure and food insecurity. This made the government of Ghana declared a state of emergency and called for external assistance to support northern Ghana. So to say that 2014 was worse is problematic. In fact, official documents show 2007 as the worse crop failure year. Authors should See many works such us Yiran and String 2016; Akudugu et al., 2012 and other related works. Also records from MOFA and NADMO can help. Their results might be as a result of memory loss, but authors needed to verify their response from official records and correlate with other works.

#3. The data was collected in 2014 and being published in 2022. That seems a bit old. This have changed and authors needed to at least go to the field to do rapid qualitative data collection to beef up. They are going to make recommendation which might not work since the data is not current.

#4. Also, some of the findings need some tweaking as there seem to be conflicting statements. For example, there is a negative association between age and reduction in consumption. This means as age increases, reduction in food consumption decreases. My knowledge of the area and the practice of the people seem opposite. This is because the youth have diverse ways of surviving while the aged due to low strength have limited options to survive.

#5. Also, Although the aged might not migrate, their children migrate and send them remittances. Remittances are very important coping or adaptive strategies and should be considered in the analysis to have a holistic view of the issues. Many of the youth are outside the region and send remittances to those left at home to buy inputs or food.

Conclusion

Conclusion is good but will need modification taken the above revisions into consideration.
