# Supplementary material for: Responding to harvest failure: Understanding farmers coping strategies in the semi-arid Northern Ghana
Source: PLoS One. 2023 Apr 14;18(4):e0284328. doi: 10.1371/journal.pone.0284328 (PMC10104303; doi:10.1371/journal.pone.0284328)
Supplement: Attachment — Submitted filename: Response to Reviewers.docx [file pone.0284328.s005.docx]

12/12/2022

Dear Editor,

The authors are very grateful to the two Reviewers for the helpful comments, response to which has helped to improve the manuscript. Below are the authors response to the respective comments made and issues raised. Thank you

Your faithfully,

Boansi

| Reviewers | Comment | Response |
| --- | --- | --- |
| Reviewer 1 | The authors did not justify the use of Poisson regression in determining the intensity of coping strategies instead of a Zero Truncated Poisson regression. The data as presented in figures 4A and B showed that all the 300 farmers adopted one form of coping strategy or another meaning that there is no zero value, hence using a zero truncated Poisson regression should be a more robust tool rather than Poisson regression | The authors agree with the reviewer on the need to use one of the truncated Poisson regression forms. However, due to the potential for overdispersion in count data, as against the assumption of equi-dispersion by regression models using Poisson distribution (Poisson, zero-truncated poisson regression and the generalized Poisson), a zero-truncated negative binomial regression model is used in determining the intensity of coping strategies in this revised manuscript. |
|  | The sampling should be elaborated further to provide understanding and ease of replication. For instance, the list of farm households from the ministry of food and agriculture does it cover the 13 districts of Ghana, and how was the systematic selection implemented? Was it the 5th or 10th... occurrences etc that were selected? | This has been addressed in the revised manuscript |
|  | The conclusion (from lines 561 – 563) that the “findings from the study thus suggest that asset-based theory may not necessarily hold since the farm households depended on the liquidation of livestock and farm implements as rapid coping responses to harvest failure” is not informed by the study. What do you mean by “asset-based theory”? this was not discussed earlier in the study. Assuming you are referring to "Asset-based welfare theory, in that case. your conclusion appears to be the opposite. The data presented showed that 94 percent of the farmers relied on assets as a coping strategy. | The conclusion has been revised and this statement deleted |
|  | It will be informative if the researchers explained how they corrected for the potential effect of a similar experience in the preceding year instead of simply stating it (Ln. 347). | We did not just state it, but included a dummy for similar experience in the preceding year (“*Severe yield loss also in 2013*”) in our analysis to control for the effect |
|  | If the experience of harvest failure focused on experiences between the years 2005 to 2014 as stated in line 351, how do you relate the socioeconomic characteristics collected between February and May 2015 (Ln. 347) to an experience and response which occurred in 2007. For instance, a farmer might have experienced crop failure in 2007 and adopted a coping strategy the same year, however, acquired land in 2014, how do you justify that coping response adopted in 2007 or 2008 is determined by land ownership (Ln 471), the same goes for other socioeconomic variables. | As stated in lines 344 to 347 of the reviewed/old version of the manuscript, “*Although farmers reportedly coped with harvest failure in different years, emphasis is placed in this study on their response to harvest failure in the year 2014, correcting however for the potential effect of a similar experience in the preceding year*”  While we document farmers’ experiences of harvest failure (just to provide some insight), we focus solely in this study on how farmers actually coped with the shock in 2014 and controlled for the effect of a similar experience from the previous year (2013) with a dummy. We are not linking the experience from 2007 with the farm household characteristics in 2014  Thus, we only link the household characteristics in 2014 with coping strategies to a shock in 2014 and correct only for the potential effect of a shock from 2013 |

| Reviewers | Comments | Response |
| --- | --- | --- |
| Reviewer 2 | Authors trying to justify their study asserted that most studies concentrate on adaptation or adaptive strategies leaving out coping strategies. This might not be true as there a lot of studies that have looked at coping strategies in the area. Also, the variables measured by authors as coping strategies are the same measured in the studies, they are saying have concentrated on coping strategies. So it might be a matter of semantics. Therefore, authors need to revisit the problem statement and identify clearly what they are trying to do. | What we stated in the reviewed manuscript, is that relative to studies that look at adaptation to shocks, the ones that focus on coping strategies (as conceptually defined in the manuscript) are few and those that look at farmers coping strategies hardly look at the determinants of the choice and intensity of the strategies. |
|  | In the study area description, authors use statistics without making references to them (see line 326 and 327). These statistics are from other works and must be cited. | The statistics from the two lines are directly from Figure 2 (authors construct based on rainfall data from NASA’s climatological database) and not previously published article |
|  | The methodology has many flaws. First of all, in lines 273 and 274, another state that both qualitative and quantitative methods are used and yet, throughout the work, no qualitative method nor results presented. What qualitative data were collected? What qualitative method was used and how were the qualitative data analysed?  The selection of the districts was skewed. All districts selected are in the central part of the region. With the exception of the Kasenas, the tribes occupying these districts virtually have the same cultural practices. However, the West (Bulsas) and the East (Kusasis and Mamprusis) are left out. Consideration of tribe/ethnicity is very important as culture affect the kind of adaptive strategies or coping strategies. Why did they concentrate on the middle? There should be a justification. Authors should note these East and West districts are the food basket of the region and any study of this kind should not leave them out. Also, the simple random and systematic selections are not described in detail. How was the simple random sampling done? How was the systematic Selection done? How many communities in each district was selected and why? Were these communities also selected by the simple random or systematic? How many farmers in each community were selected and why? All these questions need to be addressed in the methodology. | The qualitative approaches used include the presentation of figures as shown in Figures 3A and 3B, 4A and 4B. In addition to these, we present some cross-tabulations in the revised manuscript.  As stated in the methodology, the districts were randomly selected and not purposively (the latter of which could have led to the selection of the ‘food basket’ as stated by the reviewer). After the random selection, experts (mainly from the regional MoFA office and the district offices) were consulted to be sure the selected districts were valid/appropriate for a broader study by the corresponding author (for his PhD between 2013-2019– on “Extreme weather events in the Sudan Savanna region of West Africa: Agricultural impacts and adaptation”). The data used for the current study is a subset of a bigger dataset used by the corresponding author for his PhD. The selected districts were, as of the time, deemed appropriate by the experts, hence we proceeded with the selection. Details however on the random and systematic selections are provided in the revised manuscript. |
|  | There are equations in the methods, but the results are not presented to reflect results from the equations. Most of the presentations can be done with cross-tabulation and that will show clear relationships than the results presented. | It would have been more appropriate for the reviewer to indicate which equations he is referring to to guide the revision, since we believe all relevant results were presented in the reviewed manuscript. We have however included some cross tabulations also in the revised manuscript, which we are sure may address this concern. |
|  | Authors report that between 2005 and 2014, the year 2014 was the year with most crop failure. I turn to disagree with this because crop failure depends on many factors including climatic events such as floods and droughts. In 2007, the entire northern Ghana recorded the worst floods leading to the worse crop failure and food insecurity. This made the government of Ghana declared a state of emergency and called for external assistance to support northern Ghana. So to say that 2014 was worse is problematic. In fact, official documents show 2007 as the worse crop failure year. Authors should See many works such us Yiran and String 2016; Akudugu et al., 2012 and other related works. Also records from MOFA and NADMO can help. Their results might be as a result of memory loss, but authors needed to verify their response from official records and correlate with other works. | While we stand to be corrected, we don’t recall stating that the year 2014 was the year with most crop failure (which by conceptual definition is subjective), but that each of the respondents for this study reported to have experienced crop failure in the year 2014, with some also stating the experience of similar shock in 2013 and other years. The years 1983 (drought), 1999 (flooding), 2005 (drought) and 2007 (flooding) are well known in terms of extreme weather events in the study area, but our interest was in documenting from farmers perspective their recent experiences of crop failure, and how they coped in the year 2014 ((*after earlier indications of bad weather*) in specific) |
|  | The data was collected in 2014 and being published in 2022. That seems a bit old. This have changed and authors needed to at least go to the field to do rapid qualitative data collection to beef up. They are going to make recommendation which might not work since the data is not current. | In as much as we agree with the reviewer that working with a more recent data would be very helpful, we disagree that recommendation made based on analysis with the current data might not work. Analysis is made with historical data to guide the drafting of appropriate policy to enhance resilience against future shocks, and this is not forbidden in empirical studies. Besides, given recent projections of a potential onslaught in future incidences of crop failure in sub-Saharan Africa, findings from this study could guide the implementation of policy measures to reduce the adoption of costly/erosive coping mechanisms. While the use of a panel data set would be preferable, we treat the use of data from only 2014 as a limitation since this dataset is from a bigger dataset gathered by the corresponding author in 2015. |
|  | Also, some of the findings need some tweaking as there seem to be conflicting statements. For example, there is a negative association between age and reduction in consumption. This means as age increases, reduction in food consumption decreases. My knowledge of the area and the practice of the people seem opposite. This is because the youth have diverse ways of surviving while the aged due to low strength have limited options to survive. | We agree with the reviewer that his/her view should have been the case and this may be supported by theory as well. But the empirical result shows otherwise. Most of the aged who may have farmed for a long time and accumulated enough assets may be able to liquidate some to smooth consumption. On health ground, reducing consumption may also not be an appropriate option for the aged. This among many other reasons could have caused this. |
|  | Also, Although the aged might not migrate, their children migrate and send them remittances. Remittances are very important coping or adaptive strategies and should be considered in the analysis to have a holistic view of the issues. Many of the youth are outside the region and send remittances to those left at home to buy inputs or food. | Among the coping strategies reported in Figure 4A is ‘Relying on family members abroad for remittances’ (*where abroad refers to members outside the immediate community*) and this strategy was used by only 4.3% of the households. However, to capture the potential effect of access to remittances on the adoption of the other coping strategies, we have included a variable on ‘*Number of family members abroad*’ in the independent variables list in the revised manuscript. |
